# Surgical Orthodontic Treatment of a Patient Affected by Type 1 Myotonic Dystrophy (Steinert Syndrome)

**DOI:** 10.1155/2017/7957961

**Published:** 2017-05-31

**Authors:** Laura Cacucci, Beatrice Ricci, Maria Moretti, Giulio Gasparini, Sandro Pelo, Cristina Grippaudo

**Affiliations:** Università Cattolica del Sacro Cuore, Largo A. Gemelli 8, 00168 Rome, Italy

## Abstract

Myotonic dystrophy, or Steinert's disease, is the most common form of muscular dystrophy that occurs in adults. This multisystemic form involves the skeletal muscles but affects also the eye, the endocrine system, the central nervous system, and the cardiac system. The weakness of the facial muscles causes a characteristic facial appearance frequently associated with malocclusions. Young people with myotonic dystrophy, who also have severe malocclusions, have bad oral functions such as chewing, breathing, and phonation. We present a case report of a 15-year-old boy with anterior open bite, upper and lower dental crowding, bilateral crossbite, and constriction of the upper jaw with a high and narrow palate. The patient's need was to improve his quality of life. Because of the severity of skeletal malocclusion, it was necessary to schedule a combined orthodontic and surgical therapy in order to achieve the highest aesthetic and functional result. Although therapy caused an improvement in patient's quality of life, the clinical management of the case was hard. The article shows a balance between costs and benefits of a therapy that challenges the nature of the main problem of the patient, and it is useful to identify the most appropriate course of treatment for similar cases.

## 1. Introduction

Myotonic dystrophy, or Steinert's disease, is the most common form of muscular dystrophy that occurs in adults [1]. This multisystemic form involves the skeletal muscles but affects also the eye, the endocrine system, the central nervous system, and the cardiac system [2]. Myotonic dystrophy is an autosomal dominant disease affecting both sexes equally, and each child of an affected person has a 50% risk of being himself affected by the disease. The genetic alteration of the disease is an abnormal expansion of CTG triplet, located in the DMPK gene (Dystrophic Myotonic Protein Kinase) on chromosome 19 [3–7]. This is the most common form of dystrophy in the adult population, whose prevalence is calculated to be about 1–10 cases per 100.000 births [8].

Steinert's disease is characterized primarily by the myotonic phenomenon, which is a continued constriction of a skeletal muscle after a voluntary or inducted constriction. The myotonia is related to an abnormal state of excitability in the muscle fiber membrane [9]. Another distinctive feature of the disease is the muscular strength deficit and the depletion of muscle mass trending slowly progressive. The strength deficit has characteristically a distal distribution (i.e., hands, forearms, feet, and legs, in particular the portion of the limb between the knee and the foot). Mimic muscles of the face are involved too, with atrophy of the temporal and masseter muscles, as well as eyelid ptosis. The use of ultrasound and electromyography demonstrated the involvement of the masseter and temporal muscles [10, 11]. Instead, the involvement of the pterygoid muscles has never been proved. Malocclusion problems are related to masticatory muscles involvement [12–16]. The trend of the loss of strength is gradual and involves in a second moment proximal districts too, but the progression varies greatly from person to person, following the expansion of CTG triplet. The weakness of the diaphragm and the alveolar hypoventilation, which cause chronic bronchitis and bronchiectasis, are common manifestations of the disease, as cardiac abnormalities; the latter are mostly due to a disorder of the cardiac conduction system, which causes bradycardia and increased PR interval. Patients with extreme bradycardia or with a high degree of atrioventricular block may die suddenly; the insertion of a pacemaker is recommended for these subjects. The mitral valve prolapse and dysfunction of the left ventricle are less frequent. The disease progresses slowly, with gradual involvement of the proximal muscles of the limbs and trunk muscles.

The age of onset and clinical manifestations are highly variable, depending on the type of genetic alteration. Marked differences are noticed in individuals and in the various members of the same family.

Some years ago, the detection of a form clinically very similar to myotonic dystrophy, but with proximal strength deficit and different genetic basis, resulted in the introduction of DM1 symbol to indicate Steinert's disease and DM2 to indicate this other form, also known as PROMM (Proximal Myotonic Myopathy). Other subjects with similar clinical characteristics did not show the alteration of the DM1 gene nor that of DM2. DM1 or Steinert's dystrophy is caused by the gene defect of myotonin protein kinase (DMPK), located on chromosome 19q13.3. The DM2 is rarer and it is secondary to the defect of the Zinc Finger Protein Gene 9 (ZNF9), encoded by chromosome 3q21. There is still a third form (DM3), which, however, does not have a precise genetic and molecular characterization.

Both the forms of dystrophy are characterized by excessive repetition (“babbling”) of a sequence of nucleotides (triplets) that in normal subjects is repeated for a limited number of times. In the subjects affected by the disease, these base sequences (CTG for DM1 and CCTG for DM2) may be repeated from tens to thousands of times, compromising the function of the gene. In general, the greater the expansion of nucleotides is, the more serious the clinical expression of the disease is. The expansion can vary in different tissues of the same individual, which explains the different manifestations of the disease.

In Steinert's myotonic dystrophy, the muscular involvement is especially evident in the distal districts (forearm, hand, leg, and foot) and the mimic muscles of the face, with a reduction in the expression movements of the face and drooping eyelids (ptosis). This disease affects the entire skeletal musculature with generalized weakness and easy fatigability. In PROMM, however, the muscle involvement prevails in districts proximal limbs (shoulders, arms, pelvis, and thigh). Both forms are characterized by myotonic phenomenon [17].

Severe congenital forms of DM1 exist: childhood and adolescence related forms and adult forms, which are the most common. In one household, different forms can coexist too, without obvious symptoms (subclinical).

People with myotonic dystrophy affecting facial and masticatory muscles have a characteristic facial appearance: long face, gonial angle, and mandibular divergence increase, which are frequently associated with malocclusions. In particular, these individuals have a constriction of the palate and an anterior open bite [15, 16]. Very often, severe dental malocclusions create discomfort in oral functions and in particular in chewing, breathing, and phonation [18, 19], besides constituting a social disadvantage [20]. In addition, the functional discomfort creates difficulty in swallowing and promotes general dental problems related to dry mouth due to the labial incompetence and mouth breathing with plaque buildup and gingivitis and greater susceptibility to caries [21].

In the paper, we present the case report of a patient with Steinert's syndrome treated with orthodontic-surgical therapy. Although therapy caused an improvement in patient's quality of life, the clinical management of the case was hard. The case report is aimed to show a balance between costs and benefits of a therapy that challenges the nature of the main problem of the patient, and it is useful to identify the most appropriate course of treatment for similar cases.

## 2. Case Presentation

### 2.1. Diagnosis

**Figure 1:**
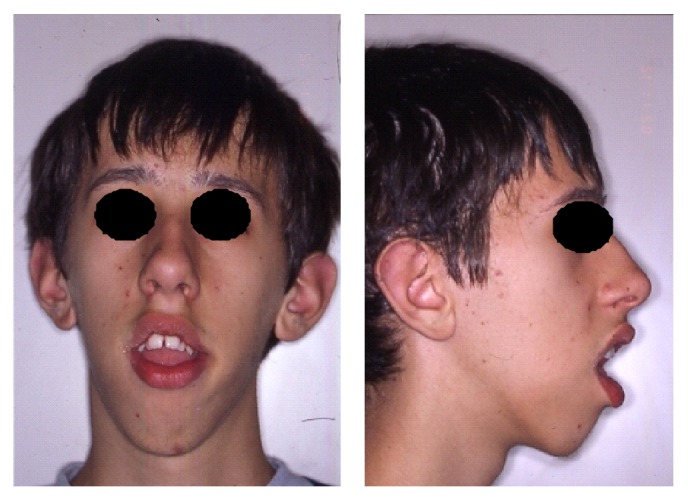
Front and lateral photographs of the patient at the time of the first visit.

The patient M. M. (male), affected by myotonic dystrophy type 1, came to our attention at the age of 15 years and seven months because he was not satisfied with his occlusion and his facial appearance. Because of lip incompetence, the patient had difficulty in eating and swallowing and difficulty in pronouncing certain phonemes. Steinert's syndrome was diagnosed when the patient was 7 years old and he presented with macroglossia, heart problems, mouth breathing, and frequent pneumonia. Genetic analysis showed a class 2 expansion of about 660 CTG. The family history revealed that the father, who died because of cardiac complications, has been suffering from myotonic dystrophy, diagnosed at age of 50, due to heart problems and difficulty in grasping objects and deficit of strength in hands. The grandmother and the paternal aunt were suffering from myotonic dystrophy too, while the sister was not affected. Physical examination showed a patient with long, narrow face, mild bilateral ptosis, lip incompetence, and hypotonia of the perioral muscles. The examination of the profile revealed a convex profile with increasing gonial angle (Figure 1) (informed consent has been obtained by the patient for publication).

**Figure 2:**
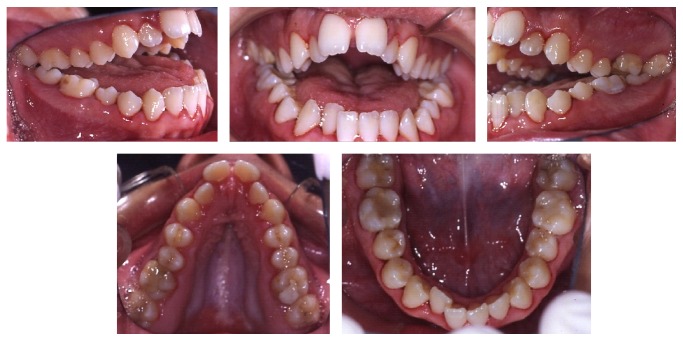
Intraoral photographs at the time of the first visit.

The intraoral examination revealed a significant anterior open bite (OVB −25 mm, OVJ 5 mm), upper and lower dental crowding, bilateral crossbite, and constriction of the upper jaw with a high and narrow palate (Figure 2).

**Figure 3:**
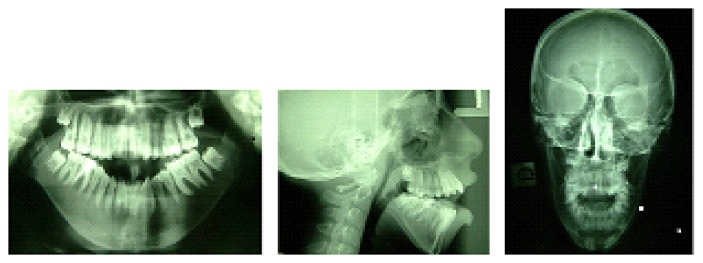
Rx before treatment.

The Illinois cephalometric analysis (Table 1) revealed a second hyperdivergent skeletal class with mandibular retrusion, tendency of vertical growth, and reduced inclination of lower and upper incisors (Figure 3).

### 2.2. Therapy

**Figure 4:**
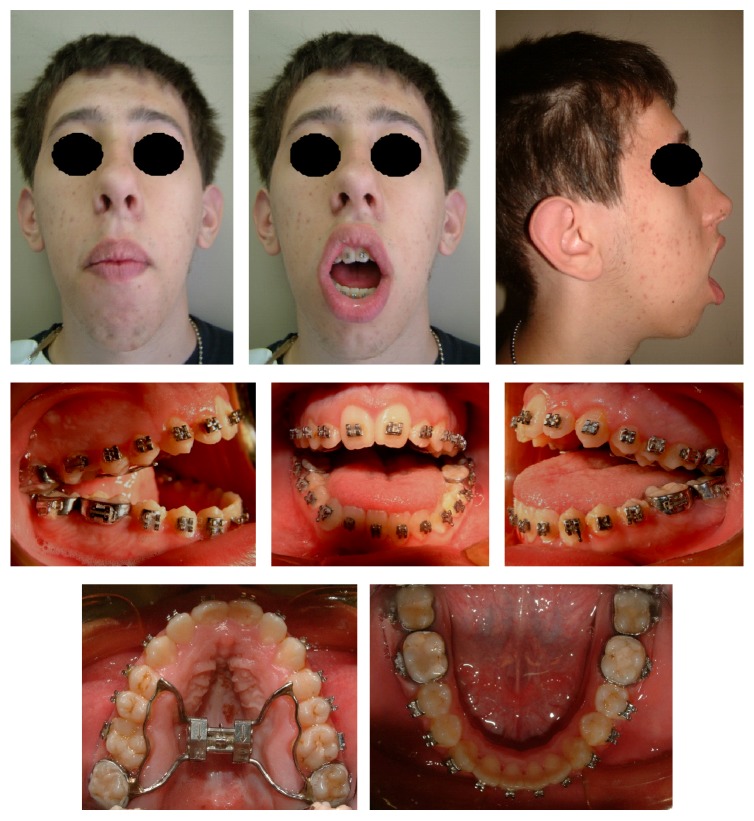
Extraoral and intraoral photographs before surgery.

**Figure 5:**
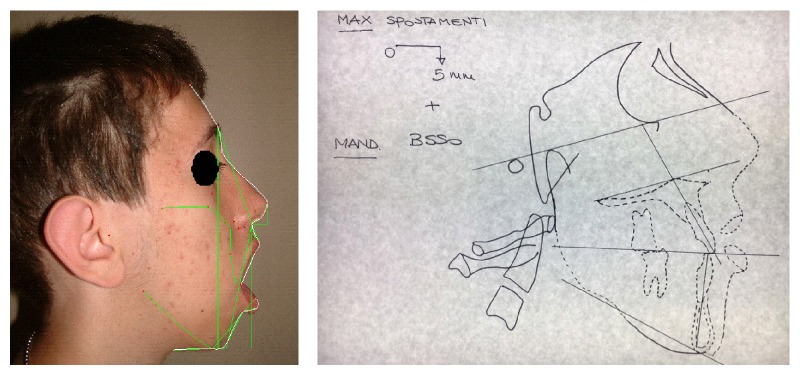
Arnett-Bergman soft tissues analysis and VTO before surgery.

Because of the severity of skeletal malocclusion, the patient received a combined orthodontic and surgical therapy in order to achieve the highest aesthetic and functional result. Maxillary rapid palatal expander performed initially two rapid expansion cycles. When the expansion was completed, an orthodontic fixed multibrackets appliance was applied in upper and lower arch. The presurgical therapy had the purpose to improve the shape of both arches, which appeared ovoid and provide a correct expansion of the upper jaw in preparation for orthognathic surgery (Figure 4). Having completed the phase of orthodontic presurgical treatment, the patient underwent maxillofacial surgery. Arnett-Bergman soft tissues analysis [22, 23] (Table 2) and surgical visual treatment objects (VTO) were performed before surgery (Figure 5). A maxillary clockwise rotation of 5 mm and BSSO of the mandible were planned. A counterclockwise rotation of 18° of the occlusal plane was planned. On VTO, a value of SNA of 77°, SNB of 73°, ANB of 5°, IMPA of 72°, and FMA of 48° were planned. On the postsurgery cephalometric analysis, we had a value of SNA of 81°, SNB of 76°, ANB of 5°, IMPA of 73°, and FMA of 49°. Therefore, the results were similar to those planned.

**Figure 6:**
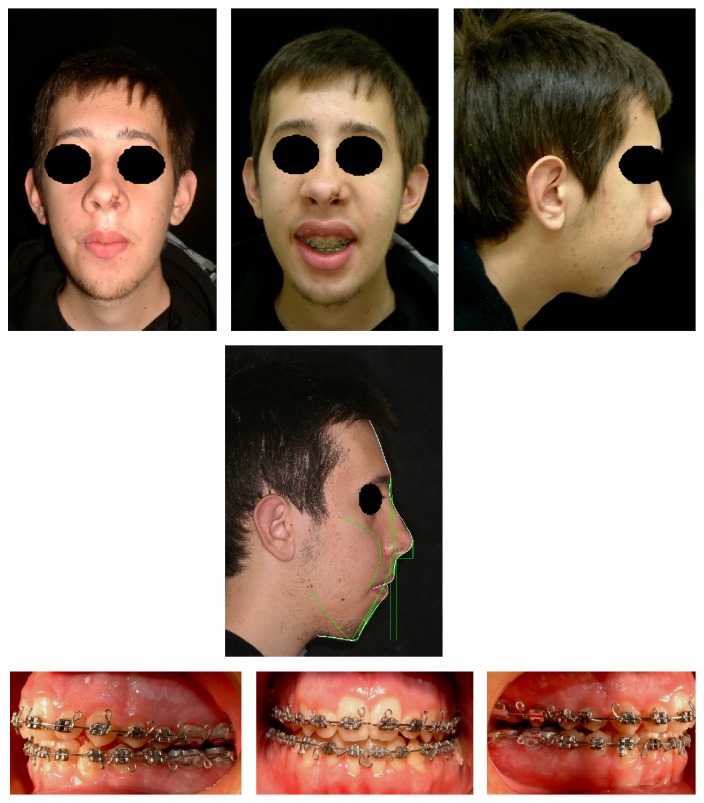
Extraoral and intraoral photographs and Arnett-Bergman soft tissues analysis after surgery.

**Figure 7:**
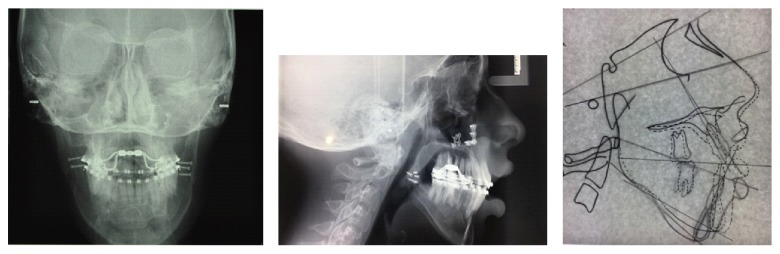
Rx after surgery.

Before surgery, the patient underwent the extraction of the third lower molars in bone inclusion. After the healing of mucosal wounds and after the finding of bone healing of extraction sites using orthopanoramic Rx, the patient had the orthognathic surgery intervention, which consisted in maxillary clockwise rotation and sagittal mandibular osteotomy repositioning in maximum intercuspidation of the dental arches. Photos (Figure 6 and Table 3) and Rx (Figure 7 and Table 4) show a facial and skeletal harmony improvement.

**Figure 8:**
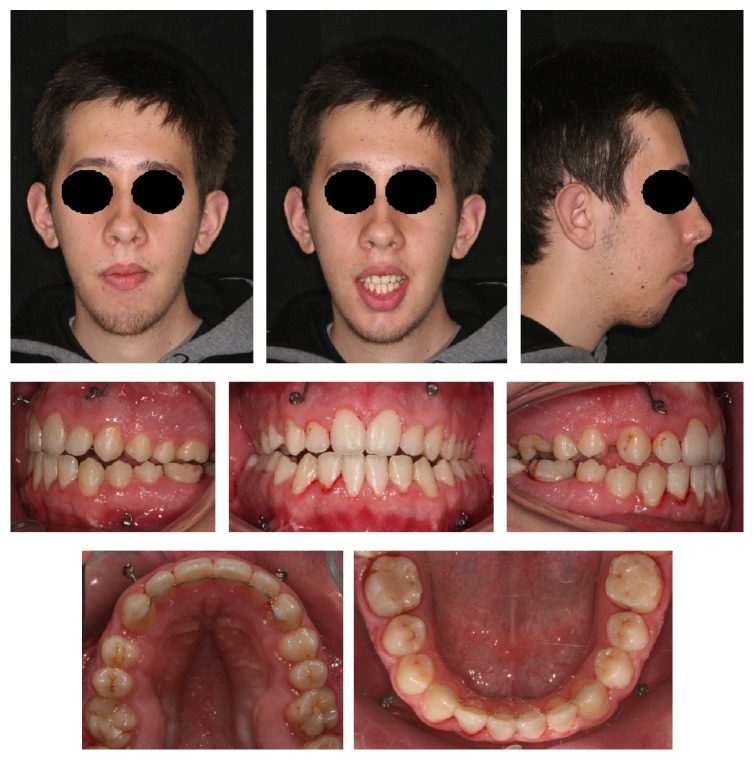
Extraoral and intraoral photographs after orthodontic therapy.

The recovery after the operation was difficult because of an extreme muscle weakness after surgery, and this is the reason why the patient avoids the orthodontic checks for two months. After surgery, orthodontic treatment completed the alignment and coordination of the dental arches. Intermaxillary elastics acted to optimize vertical occlusal relationships. In this phase, the rapid maxillary expander has been kept in place to prevent the relapse of the maxillary constriction. Because of an initial relapse of the open bite, the expander was removed, and the patient began speech therapy to improve the posture of the tongue. Six months later, there was a recurrence of the palatal constriction. One year after surgery, Bollard screws were positioned to attach vertical elastics to fixed retainers in order to counter the open bite. Two years after surgery, the fixed orthodontic appliance was removed and upper and lower Schwarz appliances of restraint were delivered to the patient to keep the result in combination with fixed upper and lower retainers (Figure 8). The patient was subjected to regular checks, until after seven months after the removal of orthodontic appliances, when it was decided to hand over to the patient two thermoformed appliances (upper and lower) with box shaped elastics positioned on frontal teeth, because of the partial relapse of open bite due to facial muscle deficits linked to the primary disease. Today, the patient periodically comes to visit, to minimize the recurrence of the open bite and to motivate the cooperation in wearing physical containment and to maintain oral hygiene.

### 2.3. Discussion

Myotonic dystrophy is a very complex molecular pathology, with multisystemic involvement [2–9]. People with myotonic dystrophy type 1 frequently have a characteristic facial appearance, such as that observed in the patient described in this paper [12, 13, 24].

Kiliaridis et al. [15] and Mercier et al. [16] claim that craniofacial malformations in patients with myotonic atrophy are associated with malocclusions in patients who have a precocious involvement of the muscles. The patient described in the case shows a constriction of the upper jaw, narrow face, increased mandibular angle, and a vertical facial growth, which are perhaps the result of increased neuromuscular function, as reported by Staley et al. [12]. The patient presents anterior open bite, as frequently found in the pathology, as reported by Portelli et al. [13] because of the prevalence of the force of gravity on the elevator muscles. Kiliaridis and Katsaros [25] say that the increasing of the vertical growth is due to an alteration of the perioral muscles in combination with a minor involvement of suprahyoid muscles. So, a new situation is established around the teeth, which alter the transversal relationships. The tongue in a lower position is not able to counterbalance the forces developed during the lowering of the lower jaw caused by the elongated facial musculature. This situation, as Kiliaridis and Katsaros [25] say, can also affect the vertical size, decreasing the width of the palate and causing a posterior crossbite. The lowered position of the mandible, in combination with the reduction of the occlusal forces, can allow a supereruption of the posterior teeth, with increased height of the palatal vault and the development of an anterior open bite.

With regard to the surgical therapy, as Manzon and Philbert [24] say, although the induction of general anesthesia is extremely safe in a healthy population, severe reaction to anesthetic and neuromuscular paralyzing agents in a myotonic patient can cause organ failure, myocardial infarction, and respiratory failure. Therefore, given the complexity of the case and the disease, a polispecialistic collaboration, comprising maxillofacial surgeons, anesthetists-resuscitators, neurologists, pulmonologists, and cardiologists, is required in the management of postoperative moment, in order to prevent pulmonary and cardiac complications. In order to perform a correct postoperative monitoring, the patient described in the case report spent the first night after surgery in a postoperative intensive care unit. The danger of complications related to general anesthesia makes this precaution strongly advisable in all patients who have to undergo surgery with general anesthesia.

Surgical movements of the skeletal bases must be programmed in order to provide the greatest postoperative surgical stability in the long term, because there is a high risk of relapse because of the progressive weakness of the temporal muscle. The weakness of the masticatory muscles can lead to recurrence of the anterior open bite closed by surgery [26, 27]. In our experience, in favor of combined orthodontic-surgical therapy, there is the improvement of oral functions and the patient's appearance. However, the high probability of recurrence, complications related to general anesthesia and the difficulties of recovery after the intervention, and the need of very long orthodontic therapy, which favor the accumulation of bacterial plaque and increase the risk of caries [28], must be kept in mind. In the case report described, in fact, throughout the orthodontic treatment, the condition of oral health deteriorated because of the buildup of plaque, resulting in gingivitis and multiple demineralization at the collar level of the teeth. Naturally, to undertake this type of very long and complex therapy, patient cooperation is necessary, but we know that, during long therapy, it always decreases [29]. These considerations lead us to assess the situation of each patient individually. It would be better to work on the prevention and early treatment of malocclusion of cooperative patients with muscular dystrophy, in order to counteract the effects of facial hypotonic muscles on occlusion and the appearance of the patient from the time of initial diagnosis of the disease [28]. Even cases successfully treated in developmental age over time may recur or worsen the functional deficit; therefore, patients should be orthodontically monitored periodically.

## 3. Conclusion

Myotonic dystrophy is a very complex multisystemic disease, in which the muscular involvement leads to a characteristic facial phenotype. The weakness of the facial muscles is the cause of malocclusions needing in adulthood orthodontic-surgical treatment. The purpose of this study was to describe the case of a patient treated with surgery and orthodontics, whose satisfaction at the conclusion of therapy demonstrated the importance of a malocclusion's therapy to provide an aesthetic improvement to the patient and a resolution of relational and functional problems. This work could be an incentive to evaluate patients with myotonic dystrophy in an early age, to try to counter the worsening of malocclusions and avoiding surgery, if possible. The propose of an early intervention may seem like a challenge, but it might be useful to identify new care pathways that make better the daily lives of these young patients and facilitate their psychosomatic development during the delicate period of adolescence.

## Figures and Tables

**Table 1:** Pretreatment cephalometric analysis.

	Case	Norm
Facial angle	78.2	88.3
Convexity angle	17.1	5
SNA	79.2	80.2
SNB	71.7	77
ANB	7.5	3.2
Mandibular angle	57.6	22.4
*y*-axis	78.9	57.3
Occlusal angle	37.6	9
Interincisal angle	121.4	129
Li-occlusal plane	8	18.4
Li-mandibular plane	−12	4.8
Ui/SN	94.6	105
Ui/A-Pg	8.7	5
Li/A-Pg	8.2	1.3
ML/SNL	63.1	/
NL/SNL	12	/
Rotational type	P1NOB	/
Growth category	2	/

**Table 2:** Arnett-Bergman soft tissues analysis before surgery.

	Case	Norm
Facial angle	149.3	165–173°
Nasal projection	12.6	13–18 mm
Nasolabial angle	98.8	94–110°
Lower face height	99	57–74 mm
Lower face%	56.2	53–56%
Upper lip length	14.9	F 18–22
		M 22–25 mm
Maxillary sulcus	126.8	127–147°
Upper lip protrusion	8.4	3 ± 1 mm
Interlabial gap	36.3	1–5 mm
Lower lip-chin length	48.4	F 43–50
		M 45–54
Mandibular sulcus	132	110–134°
L. lip protrusion	17.9	2 ± 1 mm
B'-SnPg' line	7.1	4 ± 1 mm
Lower face-throat angle	13.4	96–110°
Throat length	85.4	51–63 mm

**Table 3:** Arnett-Bergman soft tissues analysis after surgery.

	Case	Norm
Facial angle	150.3	165–173°
Nasal projection	18	13–18 mm
Nasolabial angle	130.9	94–110°
Lower face height	90	57–74 mm
Lower face%	49.3	53–56%
Upper lip length	20.9	F 18–22
		M 22–25 mm
Maxillary sulcus	168.7	127–147°
Upper lip protrusion	6.6	3 ± 1 mm
Interlabial gap	0	1–5 mm
Lower lip-chin length	66.1	F 43–50
		M 45–54
Mandibular sulcus	173.7	110–134°
L. lip protrusion	7.8	2 ± 1 mm
B'-SnPg' line	4.9	4 ± 1 mm
Lower face-throat angle	18.5	96–110°
Throat length	71	51–63 mm

**Table 4:** Postsurgery cephalometric analysis.

	Case	Norm
Facial angle	80	88.3
Convexity angle	10.3	5
SNA	81.3	80.2
SNB	75.9	77
ANB	5.4	3.2
Mandibular angle	49.3	22.4
*y*-axis	74.7	57.3
Occlusal angle	21.4	9
Interincisal angle	142	129
Li-occlusal plane	10.8	18.4
Li-mandibular plane	−17.1	4.8
Ui/SN	91.8	105
Ui/A-Pg	6.2	5
Li/A-Pg	3.6	1.3
ML/SNL	53.4	/
NL/SNL	12.8	/
Rotational type	P1NOB	/
Growth category	2	/

## Abbreviations

DM1: Myotonic dystrophy type 1
DM2: Myotonic dystrophy type 2
PROMM: Proximal Myotonic Myopathy
DMPK: Myotonin protein kinase
DM3: Myotonic dystrophy type 3
ZNF9: Zinc Finger Protein Gene 9
OVB: Overbite
OVJ: Overjet
Rx: Radiography.
